# Brain Banking in the United States and Europe: Importance, Challenges, and Future Trends

**DOI:** 10.1002/alz.090853

**Published:** 2025-01-09

**Authors:** Benjamin Danner, Angelique D. Gonzalez, William Cole Corbett, Mohammad Alhneif, Shahroo Etemadmoghadam, Julie Parker‐Garza, Margaret E Flanagan

**Affiliations:** ^1^ University of Texas Health Science Center at San Antonio, San Antonio, TX USA; ^2^ Glenn Biggs Institute for Alzheimer’s & Neurodegenerative Diseases, University of Texas Health Sciences Center at San Antonio, San Antonio, TX USA; ^3^ University of Texas Health Science Center San Antonio, San Antonio, TX USA

## Abstract

**Background:**

In recent years, brain banks have become a valuable resource for examining the molecular underpinnings of various neurological and psychological disorders such as Alzheimer’s disease and Parkinson’s disease; however, the availability of brain tissue has significantly declined. Proper collection, preparation, and preservation of post‐mortem autopsy tissue are essential for optimal downstream brain tissue distribution and experimentation. Collaborations between brain banks through larger networks such as NeuroBioBank with centralized sample request mechanisms promote tissue distribution where brain donations are disproportionately lower. These collaborations would also help standardize the donation and sample preparation processes and enable the distribution of knowledge and information amongst neuroscientists. Implementing ethical measures during donation enhances the responsible conduct of scientific studies and cultivates trust among donors. Education and outreach programs that foster collaboration between hospitals, nursing homes, neuropathologists, and other research scientists can alleviate concerns among potential brain donors. Furthermore, ensuring that biorepositories accurately reflect the true demographics of communities will result in research data that reliably represents populations. Therefore, there is a need for initiatives to enhance the sustainability and efficacy of brain banks, fostering an environment that aligns with the evolving needs of scientists and researchers.

**Method:**

This review involved a comprehensive search of Google, Google Scholar, and PubMed databases. Relevant English articles and information from websites pertaining to brain banking in the United States and Europe were extracted for thorough examination, up until November 2023.

**Result:**

Brain autopsy rates have had a steady decline from 41.1% in 1964 to less than 5% in many hospitals today. Additionally, published cost estimates of collecting a postmortem brain approximate the cost to be between $10,000 and $30,000 USD. With brain tissue scarcity posing an utmost problem to many neuropathologists and scientists, increasing standard operating procedures, centralization of Brain banks will serve to combat the decline.

**Conclusion:**

Successful implementation of these initiatives, such as enhancing collaboration between intra‐institutional and inter‐institutional brain banks alongside integration of innovative technologies and proactive efforts to improve brain donations and storage will grant scientists improved access to brain tissue. This will then facilitate a deeper understanding of the neurological diseases that impact millions.

